# Supplementary material for: The causal role of circulating inflammatory markers in osteoporosis: a bidirectional Mendelian randomized study
Source: Front Immunol. 2024 Jul 18;15:1412298. doi: 10.3389/fimmu.2024.1412298 (PMC11291241; doi:10.3389/fimmu.2024.1412298)
Supplement: Supplementary file 1 [file DataSheet_1.zip › supplementary material/OSTEOPOROSIS-CIM/GCST90274832.tsv.gz_leave_one_out.pdf]

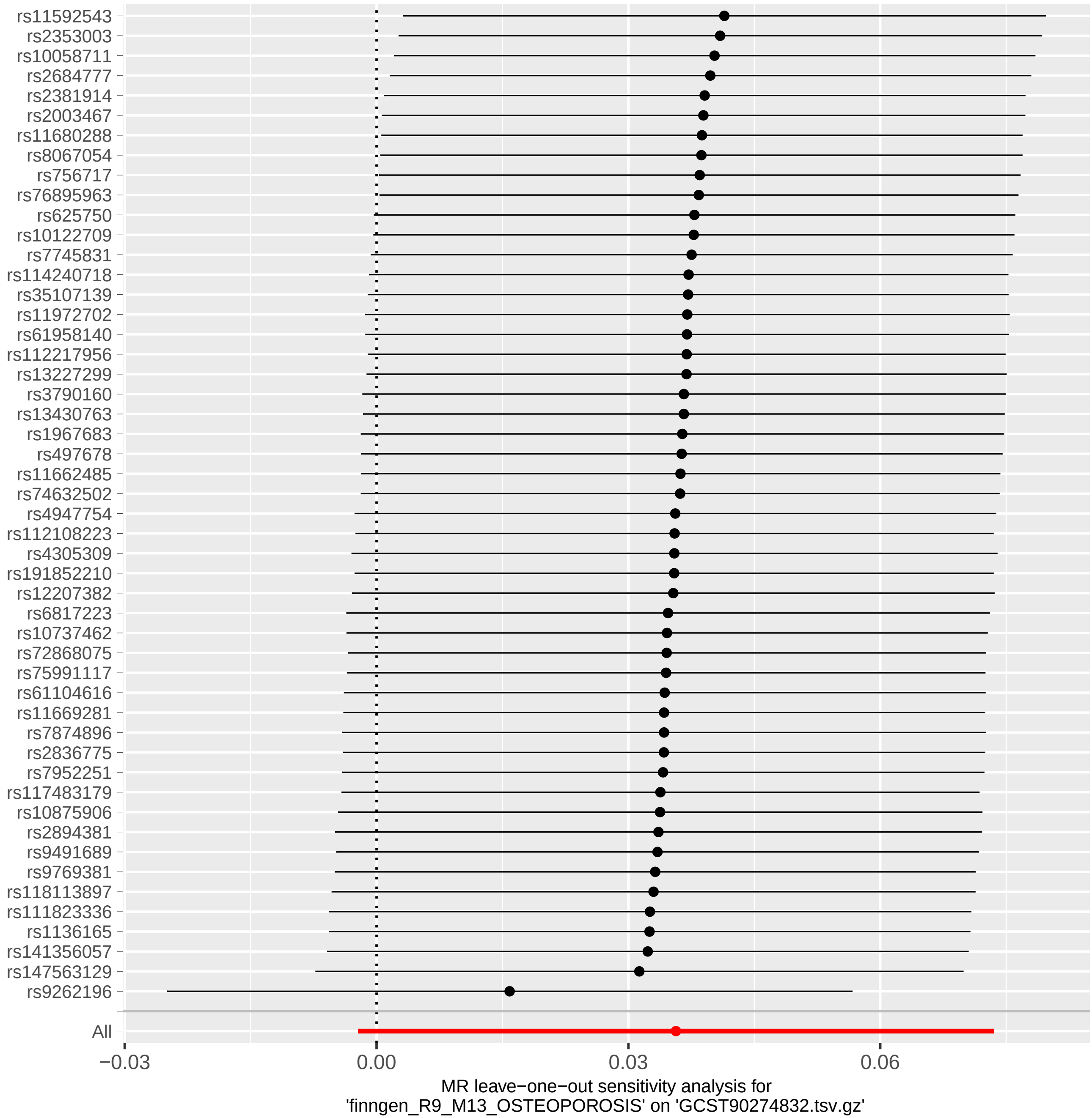
rs11592543
rs2353003
rs10058711
rs2684777
rs2381914
rs2003467
rs11680288
rs8067054
rs756717
rs76895963
rs625750
rs10122709
rs7745831
rs114240718
rs35107139
rs11972702
rs61958140
rs112217956
rs13227299
rs3790160
rs13430763
rs1967683
rs497678
rs11662485
rs74632502
rs4947754
rs112108223
rs4305309
rs191852210
rs12207382
rs6817223
rs10737462
rs72868075
rs75991117
rs61104616
rs11669281
rs7874896
rs2836775
rs7952251
rs117483179
rs10875906
rs2894381
rs9491689
rs9769381
rs118113897
rs111823336
rs1136165
rs141356057
rs147563129
rs9262196
All
−0.03
0.00
0.03
0.06
MR leave−one−out sensitivity analysis for
'finngen_R9_M13_OSTEOPOROSIS' on 'GCST90274832.tsv.gz'
